# Optimizing Extraction and HPLC‐ESI‐QTOF‐MS/MS Analysis of Bound Phenolics From Okra (*Abelmoschus esculentus*) and Their Biological Activity

**DOI:** 10.1002/fsn3.72059

**Published:** 2026-07-03

**Authors:** Bingjie Zhang, Rongjuan Liu, Hong Zhang, Ting Xv, Yue Lv, Yuan Xv, Haiqiang Chen, Hu Zhu, Xiaolin Shi, Mingyan Yan, Yinping Li

**Affiliations:** ^1^ College of Biological Engineering Qingdao University of Science and Technology Qingdao People's Republic of China; ^2^ Outpatient Department, Qingdao Central Hospital University of Health and Rehabilitation Sciences Qingdao People's Republic of China; ^3^ Department of Pulmonary and Critical Care Medicine, Shandong Provincial Key Medical and Health Discipline, Qingdao Central Hospital University of Health and Rehabilitation Sciences Qingdao People's Republic of China; ^4^ Shandong Nice Health Technology Co., LTD Jinan People's Republic of China; ^5^ Shandong Guangpu Biotechnology Co., Ltd Zibo People's Republic of China

**Keywords:** antimicrobial property, antioxidant activity, bound phenolics, extraction, HPLC‐ESI‐QTOF‐MS/MS, okra

## Abstract

Okra ( *Abelmoschus esculentus* ) is rich in phenolic compounds and shows various bioactivities. Free phenolic compounds in okra were identified, while the knowledge about its bound phenolics remains unclear. Consequently, this study aimed to optimize the alkaline extraction for okra pulp bound phenolics (OPBP) and okra seed bound phenolics (OSBP). The maximum total phenolic content (TPC) of OPBP was 7.77 mg GAE/g DW under the optimal conditions of liquid–solid ratio 60:1, 60 min, and 60°C. The maximum TPC of OSBP was 83.03 mg GAE/g DW under the optimal conditions of liquid–solid ratio 80:1, 40 min, and 70°C. Then 14 phenolic compounds in OPBP and 12 phenolic compounds in OSBP were identified by using HPLC‐ESI‐QTOF‐MS/MS, among which nine other compounds including formononetin were reported in okra for the first time. Furthermore, the ferric reducing antioxidant power (FRAP) values were 4.30 and 5.18 mmol Fe^2+^/g for OPBP and OSBP, respectively. The IC_50_ values of diammonium 2,2′‐azino‐bis (3‐ethylbenzothiazoline‐6‐sulfonate) (ABTS) radical scavenging activity were 3.34 for OPBP and 3.69 μg/mL for OSBP. Interestingly, OPBP exhibited stronger antibacterial activity against *Escherichia coli* and *Vibrio parahaemolyticus* , while OSBP was more effective in inhibiting the growth of *Staphylococcus aureus* and *Listeria monocytogenes* . These results provide valuable insights for assessing the nutritional and health benefits of okra.

## Graphical abstract

Extraction and characterization of okra bound phenolics. The extraction of bound phenolics from okra pulp (OPBP) and seeds (OSBP) was optimized, achieving maximum yields of 7.77 and 83.03 mg GAE/g DW, respectively. Using HPLC‐ESI‐QTOF‐MS/MS, multiple phenolic compounds were identified, with nine being reported in okra for the first time. Functional evaluation revealed that both OPBP and OSBP possess strong antioxidant capacities. Furthermore, they exhibited specific antibacterial activities: OPBP targeted Gram‐negative bacteria ( *E. coli* , *V. parahaemolyticus* ), whereas OSBP was more effective against Gram‐positive strains ( *S. aureus* , *L. monocytogenes* ). This work provides a theoretical basis for the comprehensive utilization of okra resources.

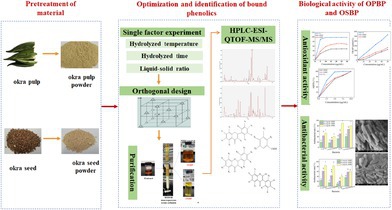

## 1 Introduction

Throughout history, the fruit, leaves, and roots of various plants have been used as natural remedies to treat numerous diseases such as inflammation, diabetes, and cardiovascular diseases (Dias et al. 2012). Crude extraction of phenolic rich plants is gaining popularity, making their chemical analysis essential for identifying the substances responsible for their health benefits (Kaur et al. 2024). Okra ( *Abelmoschus esculentus* ), a member of the *Malvaceae* family, is an annual herbaceous plant primarily cultivated in tropical and subtropical regions (Wang et al. 2023). Okra has garnered increasing attention due to its remarkable nutritional value and delightful taste. Besides, it is believed to possess potential therapeutic properties, including antidiabetic, anticancer, anti‐fatigue, antioxidant, immunoregulatory, and cardioprotective effects (Sipahi et al. 2022; Zhou et al. 2024; Li et al. 2022). The main chemical constituents of okra are mucilage, polysaccharides, and polyphenols. Especially, the polyphenols are considered the main active ingredient in okra. To date, researcher have predominantly focused on free phenolics, which are readily extractable using conventional solvents or ultrasound‐assisted extraction techniques (Arapitsas 2008; D'Urso et al. 2020; Ong et al. 2021; Wang et al. 2023). These investigations have successfully characterized a spectrum of bioactive molecules within okra, including luteolin, quercetin, rutinoside, and oligomeric catechins, alongside phenolic acids like 4‐hydroxybenzoic and ferulic acids. These investigations demonstrated that the okra polyphenols showed antidiabetic, antihyperglycemic, and antioxidant activities.

Although free phenolics in okra have been widely investigated, there have been no reports so far on the extraction of bound phenolics from okra after a quick search, to our knowledge. In various plant‐based foods, including fruits and vegetables, bound phenolics (non‐extractable polyphenols) constitute a substantial fraction (20%–60%) of the total phenolic content (Acosta‐Estrada et al. 2014). Unlike free phenolics, these bound phenolics are covalently or non‐covalently anchored to the structural components of the plant cell wall, such as cellulose, hemicellulose, lignin, pectin, and proteins (Zhang et al. 2022). Consequently, the extraction of bound phenolics normally requires the assistance of acid, alkaline, or enzyme (Dominguez‐Rodriguez et al. 2021). Except its high content, studies on other plant matrices have demonstrated that bound phenolics showed strong antioxidant and prebiotic activities, as well as the ability to effectively reduce postprandial blood glucose levels (Zhang, Wu, et al. 2023). Further, many researches certified that phenolic compounds extracted from plants can act as natural antibiotics (Efenberger‐Szmechtyk et al. 2021; Bae et al. 2022; Yemis et al. 2022; Wang et al. 2024; Keyvani‐Ghamsari et al. 2023).

In this study, bound phenolics were defined as phenolics that remained in the residue after the removal of free phenolics with aqueous ethanol and were subsequently released by 2 M NaOH treatment. Considering different part of okra may contain different chemical composition showing diverse bioactivities, a comprehensive study to investigate the bound phenolics composition of okra seed and okra pulp is necessary. And in this study okra pulp was the part of okra pod removed okra seed. Consequently, the present study focused on (1) optimizing the extraction of bound phenolics in okra pulp and okra seed; (2) identifying their composition using high performance liquid chromatography coupled with mass spectrometry (HPLC‐ESI‐QTOF‐MS/MS); (3) evaluating their antioxidant and antibacterial activity.

## 2 Materials and Methods

### 2.1 Materials

The 10 kg fresh Wufu okra (cultivar) was procured from Fujian Province, China. The pods, harvested at a length of 8–10 cm for optimal texture in January 2025, were transported to the laboratory at room temperature. The 1,1‐diphenyl‐2‐picrylhydrazyl (DPPH), diammonium 2,2′‐azino‐bis (3‐ethylbenzothiazoline‐6‐sulfonate) (ABTS), 2,4,6‐tri (2‐pyridyl)‐1,3,5‐triazine (TPTZ), gallic acid, and 2‐(3,6‐Diacetoxy‐2,7‐dichloro‐9 h‐xanthen‐9‐yl) benzoic acid (H_2_DCFDA) were purchased from Solarbio (Beijing, China). The alkaline phosphatase and adenosine triphosphate (ATP) detection kit were purchased from Biyuntian (Shanghai, China). The *Escherichia coli* (ATCC 25922), *Vibrio parahaemolyticus* (ATCC 17802), *Staphylococcus aureus* (ATCC 25923), and *Listeria monocytogenes* (NCTC 7973) were provided by Qingdao University of Science and Technology.

### 2.2 Pretreatment of Okra Pulp and Seed

The pulp and seed of fresh okra were dried at 60°C for 24 h in an oven. Dried okra pulp and seed were powered in a grinder and then passed through a 40‐mesh sieve. The conditions for free phenol removal from okra pulp and okra seed were optimized, respectively. In brief, 1 g okra pulp powder was mixed with 70% ethanol (25 mL) and then the free phenolics were removed under the conditions of 45°C, 144 W, and 45 min using an ultrasonic instrument (Wang et al. 2023). Meanwhile, 1 g okra seed powder was homogenized with 60% ethanol (70 mL) and then the free phenolics were removed under conditions of 40°C, 144 W, and 40 min. Finally, the sediment was collected after centrifuging at 4000 *g* for 5 min using a centrifuge. The pretreated okra pulp and seed powder were obtained after the sediment was dried (65°C, 12 h).

### 2.3 Single Factor Experiment for Extraction of Bound Phenolic Compounds

There is no research about the extraction of bound phenolics from okra. Based on our preliminary experiment, 2 M NaOH was used to extract bound phenolics. Then, the effect of extracted conditions on total phenolic content (TPC) was determined (Table 1). In a nutshell, the pretreated okra pulp or seed were hydrolyzed with 2 M NaOH at different liquid (mL)—solid (g) ratios (40:1–70:1 for okra pulp; 50:1–80:1 for okra seed), respectively. The effect of hydrolyzed temperatures (40°C–70°C for okra pulp; 50°C–80°C for okra seed) and hydrolyzed time (30–60 min for okra pulp; 40–70 min for okra seed) on TPC was also studied. Afterward, the extracted solutions were centrifuged (4500 *g*, 5 min), then adjusting the pH of supernatant to 6.0 with 2 M HCl.

**TABLE 1:** Single factor design for extraction of bound phenolic compounds.

Single factor	Liquid—solid ratio (X/1)	Temperature (°C)	Time (min)
From okra pulp			
Liquid—solid ratio (X/1)	40:1, 50:1, 60:1, 70:1	1:50	1:50
Temperature (°C)	50	40, 50, 60, 70	60
Time (min)	50	50	30, 40, 50, 60
From okra seed			
Liquid—solid ratio (X/1)	50:1, 60:1, 70:1, 80:1	1:70	1:70
Temperature (°C)	70	50, 60, 70, 80	70
Time (min)	60	60	40, 50, 60, 70

### 2.4 Orthogonal Design for Extraction of Bound Phenolic Compounds

To further enhance the extraction efficiency, orthogonal design approaches were employed using TPC as a dependent variable. Therefore, L9 (3^3^) test design was utilized to optimize the liquid–solid ratio (A), temperature (B), and time (C) (Table 2).

**TABLE 2:** Orthogonal test result of bound phenolic compounds.

No.	A (X/1)	B (°C)	C (min)	TPC (mg GAE/g DW)
From okra pulp				
1	40	50	50	4.19
2	40	60	60	5.81
3	40	70	70	5.50
4	50	50	60	6.04
5	50	60	70	5.37
6	50	70	50	4.67
7	60	50	60	6.47
8	60	60	50	5.93
9	60	70	70	7.12
K1	5.17	5.57	4.93	
K2	5.36	5.70	6.32	
K3	6.51	5.76	5.78	
R	1.34	0.19	1.39	
From okra seed				
1	60	50	40	67.33
2	60	60	50	65.27
3	60	70	60	72.46
4	70	50	50	62.59
5	70	60	60	72.55
6	70	70	40	78.61
7	80	50	60	71.13
8	80	60	40	71.63
9	80	70	50	79.85
K1	68.35	67.02	73.24	
K2	71.25	69.82	71.67	
K3	74.20	76.97	68.89	
R	5.85	9.95	3.28	

### 2.5 Measurement of TPC and Total Flavonoid Content (TFC)

The TPC and TFC were measured according to the protocol outlined by Xia et al. (2015). The TPC was represented as mg of gallic acid equivalent (GAE) per g of pretreated okra pulp or okra seed dry weight (DW) (mg GAE/g DW). The TFC was quantified using rutin as a reference compound and represented as mg of rutin equivalent (RE) per g of pretreated okra pulp or okra seed DW (mg RE/g DW).

### 2.6 Production of Bound Phenolics From Okra Pulp and Seed

The bound phenolics from pretreated okra pulp and okra seed were extracted under their optimized extraction conditions, respectively. The extracted solution was loaded on a D101B macroporous resin column (1.6 cm × 60 cm), then eluting with water in order to remove NaCl and other substances. The target bound phenolics were recovered by elution with 75% ethanol. This process yielded okra pulp bound phenolics (OPBP) and okra seed bound phenolics (OSBP).

### 2.7 Analysis of OPBP and OSBP by HPLC‐ESI‐QTOF‐MS/MS

Chromatographic analysis was performed using an Agilent 1260 Liquid Chromatography system (Agilent Technologies, Palo Alto, CA, USA) coupled to a micrOTOF‐Q II mass spectrometer (Bruker Daltoniks, Bremen, Germany). The OPBP and OSBP were separated using a ZORBAX Eclipse XDB‐C18 column (4.6 mm × 250 mm, 5 μm), respectively. The mobile solution was 0.1% formic acid (A) and acetonitrile (B). The gradient elution program was set as follows: 0–1.5 min, 95% A; 1.5–15.5 min, 95%–40% A; 15.5–16.5 min, 40%–5% A; 16.5–17.5 min, 5%–95% A; 17.5–21.5 min, 95% A. The column temperature was maintained at 35°C with a constant flow rate of 0.3 mL/min. The ESI source is in negative ion mode. The optimal source parameter setting follows in capillary temperature at 300°C, ion source voltage at 5 kV, and source temperature at 320°C. The maximum injection time was 100 ms. The collision mode adopts high‐energy collision‐induced dissociation with stepped energy (20, 40, and 60 eV).

### 2.8 The Antioxidant Activity of OPBP and OSBP

#### 2.8.1 DPPH Radical Scavenging Capacity Assay

The DPPH radical scavenging ability of OPBP and OSBP was determined according to the protocol described by Hu et al. (2023) with some modifications. A standard solution of 2 mM DPPH was prepared using ethanol as solvent. Mixing the standard DPPH solution with the same volume of OPBP or OSBP solution with different concentrations (5, 10, 15, 20, 25, 30 μg/mL). The mixture was incubated in darkness for 30 min, then measuring their absorbance at 517 nm. The positive control was vitamin C (V_C_). The calculating formula for DPPH radical scavenging capacity was as follows:

(1) $$\text{DPPH radical scavenging}=\left[1-\frac{A_{i}-A_{i0}}{A_{0}}\right]\times 100\%$$

*A*_0_ is the absorbance value of the DPPH solution without OPBP or OSBP; *A*_*i*_ is the absorbance value of OPBP or OSBP with DPPH solution; *A*_*i*0_ is the absorbance value of OPBP or OSBP without DPPH solution.

#### 2.8.2 Ferric Reducing Antioxidant Power (FRAP) Test

The reducing Fe^3+^ capacities of OPBP and OSBP were determined according to the report of Zhang, Su, et al. (2023). The 1.9 mL FRAP solution (37°C) was mixed with 0.1 mL OPBP or OSBP solution with different concentrations (10, 20, 30, 40, 50 μg/mL). Then their absorbance was determined at 593 nm after incubating in darkness (30 min, 37°C). The FRAP values were calculated based on the standard curve of ferrous sulfate and expressed in mmol Fe^2+^ equivalent per g of OPBP or OSBP (mmol Fe^2+^/g).

#### 2.8.3 ABTS Radical Scavenging Capacity

The scavenging capacities of OPBP and OSBP for ABTS radical were determined according to report of Chen et al. (2024). In this assay, ABTS (7 mM) and K_2_S_2_O_8_ (2.4 mM) was mixed with equal volume and then placed in the darkness for 14 h at room temperature. Then diluting the mixed solution with 40 mM sodium phosphate buffer (pH 7.4) until its absorbance at 735 nm reached about 0.7. The diluted solution was standard ABTS solution. Then 0.4 mL OPBP and OSBP solution was mixed with 2.6 mL standard ABTS solution, respectively. After 30 min dark reaction, their absorbance was determined at 735 nm. The V_C_ was used as positive control. The capacity of scavenging ABTS was calculated as follow:

(2) $$\text{ABTS scavenging}=\left[\frac{A_{0}-A_{i}}{A_{0}}\right]\times 100\%$$

*A*_0_ was the absorbance value of 2.6 mL standard ABTS liquor with 0.4 mL water; *A*_*i*_ was the absorbance value of 2.6 mL standard ABTS liquor with 0.4 mL OPBP or OSBP solution, respectively.

### 2.9 Antibacterial Mechanism of OPBP and OSBP

#### 2.9.1 The Bacterial Inhibition Rate of OPBP and OSBP

The bacterial inhibition rate of OPBP and OSBP were measured according to the research of Sun et al. (2021) with some modification. The *E. coli* , *S. aureus* , and *L. monocytogenes* were cultured in beef extract peptone broth medium (5 g/L beef extract, 10 g/L peptone, 10 g/L NaCl, pH 7.2) for 12 h at 37°C, respectively. The *V. parahaemolyticus* was cultured in sodium chloride broth medium (10 g/L peptone, 30 g/L NaCl, pH 8.0) for 12 h at 30°C. Then, the above bacteria suspension (50 μL) was added to test tubes which contained 10 mL medium and 200 μL OPBP or OSBP solution. After incubation at 37°C ( *V. parahaemolyticus* at 30°C) for 20 h, inhibitory effects of OPBP and OSBP on bacteria were assessed based on their absorbance value at 600 nm (OD_600_).

The bacteria inhibition rate was calculated as follows:

(3) $$\text{Bacterial inhibition rate}=\frac{A_{0}-A_{i}}{A_{0}}\times 100\%$$

*A*_0_ was the absorbance value of bacteria suspension cultured with medium; *A*_*i*_ was the absorbance value of bacteria suspension cultured with OSBP (or OPBP).

#### 2.9.2 The Release of Nucleic Acid and Protein

The impact of OPBP and OSBP on the release of intracellular substances was investigated based on the method reported by Efenberger‐Szmechtyk et al. (2021) with some modification. Briefly, bacteria were incubated at 37°C ( *V. parahaemolyticus* at 30°C) for 20 h, then centrifuged (4500 *g*, 5 min). The cultured bacteria cell was re‐suspended in normal saline, adjusting the bacterial cell count to 10^7^ colony forming unit per milliliter (CFU/mL). The bacterial suspension (100 μL) was added to test tubes containing 10 mL of medium and 200 μL of OPBP or OSBP solution at varying concentrations. The mixtures were incubated for 12 h at 37°C, except for *V. parahaemolyticus* , which was incubated at 30°C. The bacterial cells were removed by filtration through a 0.22 μm membrane. Then the absorbance of the aseptic solution was determined at 260 and 280 nm.

#### 2.9.3 Determination of ATP Content and Extracellular Alkaline Phosphatase Activity

The ATP content and extracellular alkaline phosphatase activity of bacterial was assessed according to the reported method with some modifications (Chen et al. 2017). Bacterial cells in the logarithmic phase were centrifuged at 4°C (2500 *g*, 5 min) to collect the cell pellet. The cell pellet was then washed three times with sterile normal saline. Then the cell pellet was re‐suspended in physiological saline (OD_600_ = 0.6). The 2 mL bacterial suspension was combined with 2 mL of OPBP or OSBP solution. The mixture was then incubated for 12 h at 37°C, except for *V. parahaemolyticus* , which was incubated at 30°C. The ATP content and alkaline phosphatase activity was processed according to the instruction of assay kit.

#### 2.9.4 Determination of Reactive Oxygen Species (ROS) Generation

The ROS generation of bacteria was assessed by fluorescent probe H_2_DCFDA according to the reported method (Dong et al. 2021). Briefly, bacteria were cultured for 20 h at 37°C ( *V. parahaemolyticus* at 30°C) and adjusted to a concentration of 10^7^ CFU/mL. Subsequently, 100 μL of the bacterial suspension was added to test tubes containing 10 mL of medium and 200 μL of OPBP or OSBP solution at varying concentrations. The mixtures were then incubated in darkness for 3 h at 37°C ( *V. parahaemolyticus* at 30°C). Subsequently, the mixture was centrifuged at 4°C for 10 min (10,000 *g*). The obtained cells were washed and re‐suspended in normal saline, and then treated with 10 μM H_2_DCFDA for 60 min. Fluorescence intensity, indicative of ROS level, was evaluated using a microplate reader. The excitation and emission wavelengths were 485 and 535 nm, respectively.

#### 2.9.5 Scanning Electron Microscopy (SEM)

The morphology changes of *E. coli* , *V. parahaemolyticus* , *S. aureus* , and *L. monocytogenes* after incubating with OSBP or OPBP was observed using SEM (JSM‐6700F; Nippon electric company, Japan) according to the research of Li et al. (2025). Briefly, 1000 μL bacterial suspensions (10^7^ CFU/mL) were mixed with 200 μL OSBP or OPBP solution (1.2 mg/mL), respectively. Then the mixture was incubated for 12 h at 37°C ( *V. parahaemolyticus* at 30°C). Bacterial cell was rinsed gently with 25 mM potassium phosphate buffer (pH 7.4) and then fixed with 2.5% glutaraldehyde. Then, samples were dehydrated using a graded ethanol series of 30%, 50%, 70%, 80%, and 90%. The fixed bacteria cell was freeze‐dried and sputtered with a layer of gold platinum. Finally, SEM images were obtained at 8 kV with 15,000× magnification.

### 2.10 Statistical Analysis

The experiment was repeated in triplicates and the result was expressed as mean ± standard deviation. Prior to statistical analysis, data normality and homogeneity of variance were verified using Shapiro–Wilk and Levene's tests, respectively. Statistical comparisons were performed using the one‐way analysis of variance with Tukey's test by SPSS 25.0, and statistical differences were considered with *p* < 0.05. The graphs were drawn by Origin 2021.

## 3 Result and Discussion

### 3.1 Optimization of Extracted Conditions for Bound Phenolics From Okra Pulp and Seed

#### 3.1.1 One Factor Design for Extraction of Bound Phenolics From Okra Pulp and Seed

##### 3.1.1.1 Influence of Liquid–Solid Ratio on the TPC

**FIGURE 1:**
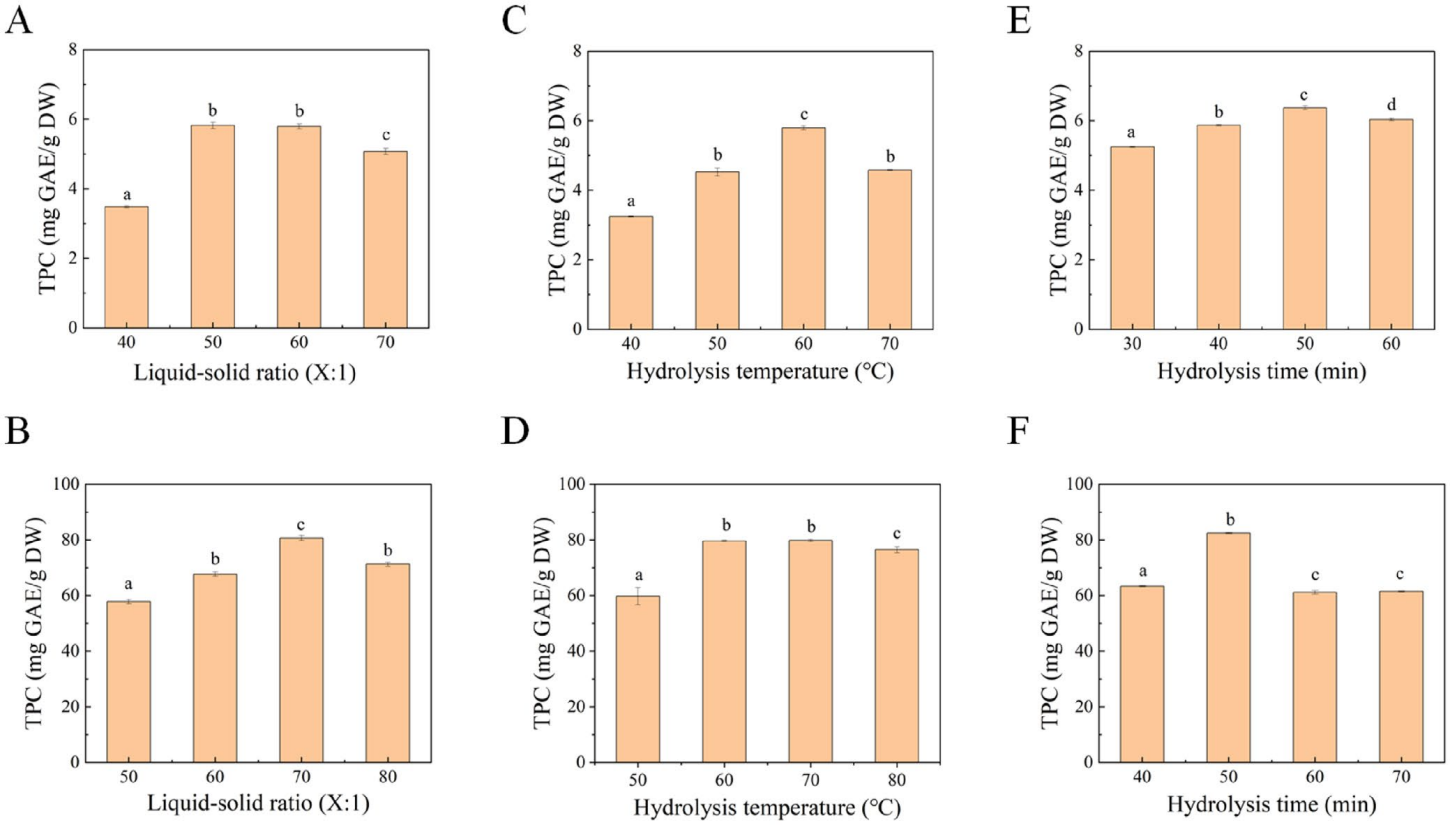
The effects of liquid–solid ratio, hydrolysis temperature, and hydrolysis time on the TPC of okra pulp (A, C, E) and okra seed (B, D, F). Data points represent means ± standard deviations (*n* = 3). Different lowercase letters indicate statistically significant differences (*p* < 0.05).

The liquid–solid ratio is an important factor that influences the efficiency and cost of extraction. As shown in Figure 1A,B, the TPC showed an upward trend as liquid–solid ratio increased until it was 60:1 (okra pulp) and 70:1 (okra seed), respectively. Further increasing the liquid–solid ratio, their TPC decreased significantly. Initially, a higher liquid–solid ratio can enhance the contact area between the sample and NaOH solution. It also reduces the viscosity of the extracted system, which is caused by the high polysaccharide content that hinders the dissolution of phenolic compounds. Nevertheless, an excessively high solvent volume absorbs more energy, resulting in insufficient energy to break the cell wall and release polyphenols, which leads to a decrease in TPC. Additionally, a larger liquid volume may contain higher amounts of dissolved oxygen, which reduces extraction efficiency, likely due to the oxidation of polyphenols (Pal and Jadeja 2019).

##### 3.1.1.2 Influence of Hydrolysis Temperature on the TPC

The TPC of okra pulp was influenced by hydrolysis temperature significantly (Figure 1C). It reached a maximum of 5.79 mg GAE/g DW at 60°C and then decreased significantly at 70°C. This aligns with the findings of Wan Mahmood et al. (2019), who reported that polyphenolic compounds degrade at temperatures exceeding 60°C. However, the TPC of okra seed showed no significant difference at 60°C and 70°C, and slight decreased at 80°C (Figure 1D). Obviously, the thermal stability of bound phenolics in okra seed is significantly higher than that in okra pulp, which may be due to their different chemical compositions. Interestingly, increasing temperature up to 80°C, the TPC of mulberry leaf still increased (Insang et al. 2022). Besides, alkaline hydrolysis itself can cause structural changes (e.g., deglycosylation, cleavage of ester bonds, possible degradation of some phenolics), which be proved by the results of HPLC‐ESI‐QTOF‐MS/MS that many flavonoid glycosides seen in free phenolics are absent in the bound fractions. These comparative results indicated that the optimal extracted temperature highly depend on the extraction method and intrinsic characteristic of material.

##### 3.1.1.3 Effect of Hydrolysis Time on TPC

When the extracted time was 50 min, the TPC of okra pulp and okra seed reached their maximum value (6.37 mg GAE/g DW and 82.51 mg GAE/g DW) (Figure 1E,F). For okra pulp, the TPC showed no significant difference (ranging from 6.37 to 6.03 mg GAE/g DW, *p* > 0.05) as the hydrolysis time increased from 50 to 60 min. In contrast, the TPC of okra seed decreased significantly (from 85.51 to 61.15 mg GAE/g DW, *p* < 0.05). This disparity is likely attributable to the higher hydrolysis temperature used for the seed (70°C) compared to the pulp (60°C). The long hydrolysis time resulted in the oxidative degradation of phenolic compounds. These results clearly demonstrated that the optimal extraction conditions for bound phenolics differ significantly between okra pulp and seed. Consequently, it was necessary to optimize the extraction condition for bound phenolics from okra.

#### 3.1.2 Orthogonal Design for Extraction of Bound Phenolic From Okra Pulp and Seed

Considering the multi‐factor interaction of extracted conditions for bound phenolic, the orthogonal design was further used to optimize the extraction conditions (Table 2). The result of variance analysis of the model was summarized in Table 3. For okra pulp, the effects of factor on TPC followed this order: factor B (hydrolysis temperature) > A (liquid–solid ratio) > C (hydrolysis time). However, for okra seed, the order of influence was factor B (hydrolysis temperature) > C (hydrolysis time) > A (liquid–solid ratio). The optimal extraction condition of bound phenolics from okra pulp was 60 min, 60°C, and liquid–solid ratio of 60:1. For okra seed, the optimal extraction condition was 40 min, 70°C, and liquid–solid ratio of 80:1. Under the optimized condition, the maximum TPC of okra pulp and okra seed was 7.77 and 83.03 mg GAE/g DW, respectively. Moreover, the TFC of okra pulp and okra seed was 2.57 and 38.70 mg RE/g DW, accounting for 33.07% and 39.96% of their TPC, respectively. Interestingly, the maximum TPC of okra seed was 10 times that of okra pulp. And this phenomenon was also reported in the extraction of free polyphenols from okra pulp and okra seed (Arapitsas 2008; Fabianová et al. 2022; Woumbo et al. 2022). Generally, the seed is storage organ for essential nutrients such as carbohydrates, proteins, and lipids. A higher content of phenolic compounds is crucial for seed dormancy and germination, as they help protect the embryo from oxidative damage. Furthermore, phenolic compounds play a role in regulating gene expression during seed germination, thereby affecting the embryo's morphological and physiological development. Moreover, flavonoids can deposit in the cell wall, thus accelerating the formation of secondary cell wall that enhances structural rigidity and resistance to environmental stresses.

**TABLE 3:** Analysis of variance for orthogonal experiments.

Factors	SS	df	MS	*F*
From okra pulp				
A	3.15	2	1.57	1.53
B	0.06	2	0.03	0.03
C	2.96	2	1.48	1.44
Error	6.17	6	1.03	
From okra seed				
A	51.33	2	25.67	0.67
B	158.19	2	79.10	2.08
C	18.93	2	9.46	0.25
Error	228.45	6	38.08	

Although no studies have specifically investigated the extraction of bound phenolics from okra, several reports have documented the extraction of free phenolics. As shown in Table 4, the content of free phenolics in okra seeds was also significantly higher than that in okra pulp. The TPC and TFC of bound phenolics from okra pulp in this study were comparable to the values reported for free phenolics from okra pulp (Wang et al. 2023; Xia et al. 2015). For okra seeds, the TPC of bound phenolics was similar to that of free phenolics; however, the TFC measured in this study was significantly higher (Woumbo et al. 2022). Furthermore, the significantly lower TPC of okra seeds reported by Xia et al. (2015) may be due to their extracted method (Xia et al. 2015). As shown in Table 4, extracting phenolics from okra seeds is more challenging than from okra pulp. However, the extraction protocol for free phenolics from seeds was identical to that used for pulp (Xia et al. 2015), which resulted in incomplete extraction from the seeds. Besides, a comparative analysis of bound phenolic content between okra and other plants was made (Wang et al. 2019; Xu et al. 2020; Tang et al. 2021). The TPC (7.80 mg GAE/g DW) of okra pulp was comparable to that of litchi pulp (7.86 mg GAE/g DW), but significantly lower than that of pitahaya pulp (11.60 mg GAE/g DW) (Xu et al. 2020; Tang et al. 2021). In contrast, the TPC (83.00 mg GAE/g DW) of okra seeds was approximately 4.4 times higher than that of raspberry seeds (18.80 mg GAE/g DW) (Wang et al. 2019). These findings indicate that okra is a good source of bound phenolics.

**TABLE 4:** Comparison of phenolic content from okra source.

Type of phenolics	Extraction condition	Source	Yield	References
Bound phenolics	2 M NaOH, 60°C, 60 min, 1:60 solid–liquid ratio	Okra pulp	TPC 7.80 mg GAE/g DW TFC 2.58 mg RE/g DW	Present study
Bound phenolics	2 M NaOH, 70°C, 40 min, 1:80 solid–liquid ratio	Okra seed	TPC 83.00 mg GAE/g DW TFC 38.70 mg RE/g DW	Present study
Free phenolics	Ultrasonic power 144 W, 70°C, 70% ethanol, 40 min, 1:25 solid–liquid ratio	Okra pulp	TPC 6.81 mg GAE/g DW TFC 2.16 mg RE/g DW	Wang et al. (2023)
Free phenolics	Microwave power of 330 W, 1:97.04 solid‐water ratio for 9.5 min	Okra seed	TPC 87.66 mg GAE/g DW TFC 2.62 mg RE/g DW	Woumbo et al. (2022)
Free phenolics	1:6 solid‐water, 100°C for 1 h (3 times)	Okra pulp	TPC 6.73 mg GAE/g DW TFC 1.02 mg RE/g DW	Xia et al. (2015)
Free phenolics	1:30 solid‐water, 100°C for 1 h (3 times)	Okra seed	TPC 29.50 mg GAE/g DW TFC 5.35 mg RE/g DW	Xia et al. (2015)
Bound phenolics	Ultrasonic power of 320 W, 2 M HCl, 85°C, 30 min, 1:10 solid–liquid ratio	Raspberry seed	TPC 18.80 mg GAE/g DW TFC 8.15 mg RE/g DW	Wang et al. (2019)
Bound phenolics	4 M NaOH, 37°C, 90 min, 1:20 solid–liquid ratio	Litchi pulp	TPC 7.86 mg GAE/g DW TFC 5.57 mg RE/g DW	Xu et al. (2020)
Bound phenolics	3 M NaOH, 30°C, 240 min, 1:30 solid–liquid ratio	Pitahaya pulp	TPC 11.6 mg GAE/g DW TFC 2.49 mg RE/g DW	Tang et al. (2021)

### 3.2 Qualitative Analysis of OPBP and OPSB

**FIGURE 2:**
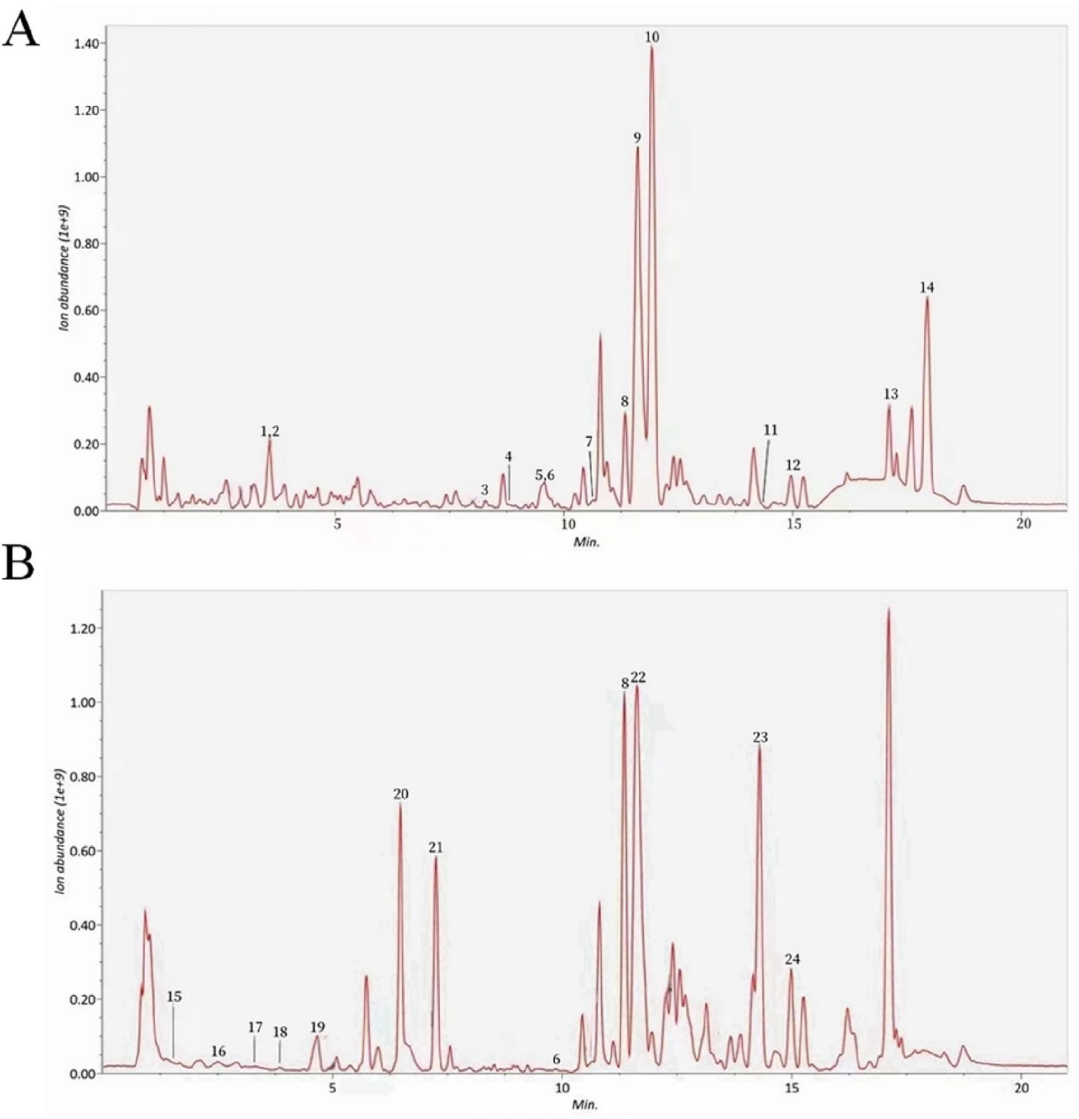
The total ion chromatogram of OPBP (A) and OSBP (B) in negative ion mode.

**FIGURE 3:**
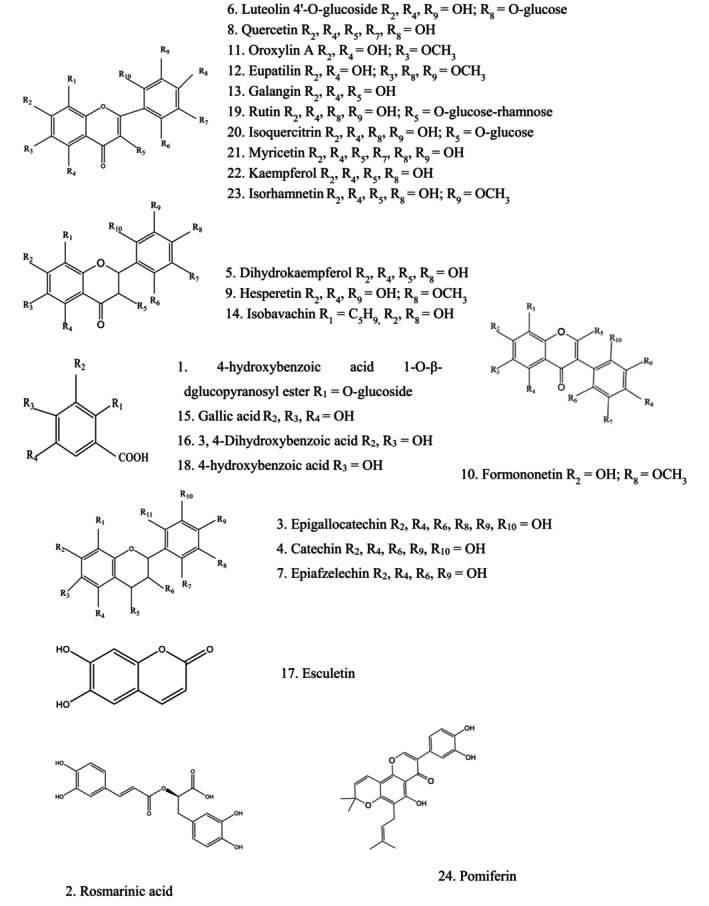
The structure of major compounds from OPBP and OSBP.

The HPLC‐ESI‐QTOF‐MS/MS was used to detect the components of OPBP and OSBP in negative ion mode (Figure 2). Identification was performed by matching precursor ion [M–H]^−^ and MS^2^ fragment peak with reference data from the database (MassBank, Respect, and GNPS) and published literature. A total of 14 bound phenolics (including 12 flavonoids and derivatives, two phenolic acids and derivatives) in OPBP and 12 bound phenolics (eight flavonoids and their derivatives, four phenolic acids and their derivatives) in OSBP were identified, respectively (Table 5). Moreover, the detailed structure of these bound phenolics was shown in Figure 3.

**TABLE 5:** Main phenolic compounds of OPBP and OSBP.

Compound	RT (min)	Formula	Precursor (*m*/*z*)	Adduct	Fragments (*m*/*z*)	Title	Confirmation mode
OPBP							
Flavonoids and their derivatives							
3	8.20118	C_15_H_14_O_7_	305	[M–H]^−^	179, 219, 221, 287	Epigallocatechin	MS/MS + database
4	8.7839	C_15_H_14_O_6_	289	[M–H]^−^	109, 123, 136, 151, 164, 179	Catechin	MS/MS + database
5	9.52021	C_15_H_12_O_6_	287	[M–H]^−^	125, 259	Dihydrokaempferol	MS/MS + literature/database
6	9.57406	C_21_H_20_O_11_	447	[M–H]^−^	133, 151, 271, 285	Luteolin 4′‐O‐glucoside	MS/MS + database
7	10.6223	C_15_H_14_O_5_	273	[M–H]^−^	229, 273	Epiafzelechin	MS/MS + literature/database
8	11.4006	C_15_H_10_O_7_	301	[M–H]^−^	107, 125, 151, 163, 179	Quercetin	MS/MS + database
9	11.8075	C_16_H_14_O_6_	301	[M–H]^−^	257, 286	Hesperetin	MS/MS + database
10	12.0521	C_16_H_12_O_4_	267	[M–H]—	108, 132, 153, 196, 252	Formononetin	MS/MS + database
11	14.4151	C_16_H_12_O_5_	283	[M–H]^−^	268	Oroxylin A	MS/MS + database
12	14.9323	C_18_H_16_O_7_	343	[M–H]^−^	270, 298, 313, 328	Eupatilin	MS/MS + database
13	16.8550	C_15_H_10_O_5_	269	[M–H]^−^	154, 155, 182, 211, 213, 239	Galangin	MS/MS + database
14	17.8801	C_20_H_20_O_4_	323	[M–H]^−^	119, 203, 221, 255	Isobavachin	MS/MS + literature/database
Phenolic acids and their derivatives							
1	3.54555	C_13_H_16_O_8_	299	[M–H]^−^	137, 93, 65	4‐hydroxybenzoic acid 1‐O‐β‐dglucopyranosyl ester	MS/MS + database
2	3.60061	C_18_H_16_O_8_	719	[2 M–H]^−^	161, 179, 197, 359	Rosmarinic acid	MS/MS + database
OSBP							
Flavonoids and their derivatives							
19	4.50675	C_27_H_30_O_16_	609	[M–H]^−^	301	Rutin	MS/MS + database
20	6.8426	C_21_H_20_O_12_	463	[M–H]^−^	301, 151, 107	Isoquercitrin	MS/MS + database
21	7.25058	C_15_H_10_O_8_	317	[M–H]^−^	151, 179, 289	Myricetin	MS/MS + database
6	9.57406	C_21_H_20_O_11_	447	[M–H]^−^	285	Luteolin 4′‐O‐glucoside	MS/MS + database
8	11.4006	C_15_H_10_O_7_	301	[M–H]^−^	107, 125, 151, 163, 179	Quercetin	MS/MS + database
22	11.64257	C_15_H_10_O_6_	285	[M–H]^−^	255, 227	Kaempferol	MS/MS + database
23	14.1884	C_16_H_12_O_7_	315	[M–H]^−^	151, 164, 271, 300	Isorhamnetin	MS/MS + literature/database
24	14.9923	C_25_H_24_O_6_	419	[M–H]^−^	375, 391	Pomiferin	MS/MS + literature/database
Phenolic acids and their derivatives							
15	1.53201	C_7_H_6_O_5_	169	[M–H]^−^	97, 125	Gallic acid	MS/MS + database
16	2.1107	C_7_H_6_O_4_	153	[M–H]^−^	108, 109	3,4‐dihydroxybenzoic acid	MS/MS + database
17	3.36221	C_9_H_6_O_4_	177	[M–H]^−^	133, 105	Esculetin	MS/MS + database
18	4.02948	C_7_H_6_O_3_	137	[M–H]^−^	93	4‐hydroxybenzoic acid	MS/MS + database

#### 3.2.1 Flavonoids and Derivative

The compound 3 ([M–H]^−^, *m*/*z* 305) showed MS^2^ peaks at *m*/*z* 179 ([M–C_6_H_6_O_3_–H]^−^), 219 ([M–C_3_H_2_O_3_–H]^−^), 221 ([M–C_4_H_4_O_2_–H]^−^), and 287 ([M–H_2_O–H]^−^). Therefore, it was tentatively identified as epigallocatechin. Compound 4 ([M–H]^−^, *m*/*z* 289) showed a fragment peak at *m*/*z* 109, indicating its B‐ring cleavage. It was provisionally detected as catechin because of its fragment peaks at *m*/*z* 123 ([M–C_6_H_5_O_2_–C_3_H_4_O–H]^−^) and 136 ([M–C_6_H_4_O_2_–C_2_H_4_O–H]^−^). Compound 5 ([M–H]^−^, *m*/*z* 287) was tentatively identified as dihydrokaempferol. It generated MS_2_ peaks at *m*/*z* 125 and 259; the former corresponds to the phloroglucinol anion formed via heterocyclic fission, while the latter corresponds to the loss of a CO molecule (Lech 2020). The compound 6 ([M–H]^−^, *m*/*z* 447) exhibited a fragment ion at *m*/*z* 285 indicating an aglycone losing. Further, compound 6 was tentatively detected as luteolin 4′‐O‐glucoside because of its other fragment peaks at *m*/*z* 133, 151, and 271. Compound 7 was tentatively characterized as epiafzelechin because of its molecular ion peak at *m*/*z* 273, which underwent sequent dissociation to yield fragment ion at *m*/*z* 229 (Ma et al. 2014). The compound 8 with molecular ion at *m*/*z* 301 was provisionally detected as quercetin due to its characteristic fragment ions at *m*/*z* 179, 163, 151, 125, and 107. Compound 19 ([M–H]^−^, *m*/*z* 609) exhibited a fragment peak at *m*/*z* 301 [M–C_12_H_20_O_9_–H]^−^ indicting the loss of rutinose moiety. Thus, compound 19 was provisionally identified as rutin. Compound 20 ([M–H]^−^, *m*/*z* 463) showed two characteristic MS^2^ ions at *m*/*z* 151 [M–C_14_H_16_O_8_–H]^−^ and 107 [M–C_15_H_16_O_10_–H]^−^, indicating two distinct pathways of the RDA reaction. Additionally, a MS^2^ ion at *m*/*z* 301 ([M–C_6_H_10_O_5_–H]^−^) indicated that compound 20 was provisionally identified as isoquercitrin. Compound 21 ([M–H]^−^, *m*/*z* 317) was provisionally identified as myricetin. This assignment was based on the observation of fragment peaks at *m*/*z* 151 [M–C_8_H_6_O_4_–H]^−^, 179 [M–C_7_H_6_O_3_–H]^−^, and 289 [M–CO–H]^−^. The compound 22 ([M–H]^−^, *m*/*z* 285) was tentatively designated as kaempferol because of its MS^2^ peaks at *m*/*z* 255 [M–CH_2_O–H]^−^ and 227 [M–CH_2_O–CO–H]^−^. Compound 23 was tentatively proposed as isorhamnetin because of its quasi‐molecular ion at *m*/*z* 315 and characteristic MS^2^ ions at *m*/*z* 151 [M–C_8_H_4_O_4_–H]^−^, 164 [M–C_8_H_7_O_3_–H]^−^, 271 [M–COO–H]^−^, and 300 [M–CH_3_–H]^−^ (Yin et al. 2019). All these compounds have been identified in free phenolics extracted from okra flower, okra pulp, and okra fruit (Arapitsas 2008; Xia et al. 2015; Deng et al. 2020; D'Urso et al. 2020; Ong et al. 2021; Woumbo et al. 2022; Yu et al. 2023; Cui et al. 2023; Wang et al. 2023). Differently, in the above references there are many dimer and flavonoid glycosides such as epigallocat dimer, catechin dimer, kaempferol 3‐O‐glucose, isorhamnetin‐3‐O‐glucoside, isorhamnetin‐3‐O‐glu‐pentoside, myricetin‐3‐O‐glucoside, myricetin‐3‐O‐glucuronide, quercetin 3‐O‐glucosyl‐xyloside, quercetin‐3‐O‐gentiobiose, and so on. However, these were not found in OPBP and OPSB. This absence is likely due to the depolymerization and deglycosylation of polyphenols resulting from sodium hydroxide treatment.

Compound 9 ([M–H]^−^, *m*/*z* 301) was tentatively identified as hesperetin because it generated fragment peaks at *m*/*z* 286 ([M–CH_3_–H]^−^) and 257 ([M–CO_2_–H]^−^). Compound 10 ([M–H]^−^, *m*/*z* 267) was tentatively detected as formononetin due to its characteristic fragment ions at *m*/*z* 108 [M–C_10_H_7_O_2_–H]^−^, 132 [M–C_8_H_7_O_2_–H]^−^, 153 [M–C_9_H_6_–H]^−^, 196 [M‐ C_3_H_3_O_2_–H]^−^, and 252 [M–CH_3_–H]^−^. Compound 11 displayed a molecular ion at *m*/*z* 283 [M–H]^−^ and MS^2^ peak at *m*/*z* 268 [M–CH_3_–H]^−^ suggesting that it was provisionally detected as oroxylin A. Compound 12 ([M–H]^−^, *m*/*z* 343) was provisionally identified as eupatilin because of its fragment peaks at *m*/*z* 298 ([M–CH_3_–CH_3_‐ CH_3_–H]^−^), 313 ([M–CH_3_–CH_3_–H]^−^), and 328 ([M–CH_3_–H]^−^). Compound 13 ([M–H]^−^, *m*/*z* 269) was tentatively identified as galangin due to its MS^2^ fragmentation peak at *m*/*z* 213 [M–C_2_O_2_–H]^−^. The compound 14 with molecular ion at *m*/*z* 323 was provisionally designated as isobavachin because of its MS^2^ peaks at *m*/*z* 255 [M–C_5_H_8_–H]^−^ and 203 [M–C_8_H_8_O–H]^−^ (Xia et al. 2022). Compound 24 ([M–H]^−^, *m*/*z* 419) was tentatively identified as pomiferin. This identification was supported by characteristic MS_2_ ions at *m*/*z* 391 and 375, which correspond to the loss of CO and CO_2_, respectively (Gajić et al. 2024). The flavonoids and their derivatives mentioned above are reported in okra for the first time, although they are widely distributed in the plant kingdom. For instance, formononetin is a major polyphenol in *Astragalus* L., while pomiferin is commonly found in *Morus* Linn. Additionally, isobavachin is a principal active component of *Psoralea corylifolia* .

#### 3.2.2 Phenolic Acids and Their Derivatives

The fragment ion at *m*/*z* 137 from compound 1 ([M–H]^−^, *m*/*z* 299) was tentatively identified as 4‐hydroxybenzoic acid. This assignment is supported by subsequent dissociation fragment ions at *m*/*z* 93 [4‐hydroxybenzoic acid–COO–H]^−^ and *m*/*z* 65 [4‐hydroxybenzoic acid–COO–CO–H]^−^. Additionally, the characteristic neutral loss of 162 Da indicated the presence of a dehydrated hexose moiety in compound 1. Consequently, compound 1 was tentatively identified as 4‐hydroxybenzoic acid 1‐O‐β‐D‐glucopyranosyl ester. Compound 2 ([2 M–H]^−^, *m*/*z* 719) was tentatively assigned to rosmarinic acid because of its fragment peaks at *m*/*z* 197 [2 M–C_18_H_16_O_8_–C_9_H_6_O_3_–H]^−^, 179 [2 M–C_18_H_16_O_8_–C_9_H_8_O_4_–H]^−^, 161 [M–C_18_H_16_O_8_–C_9_H_10_O_5_–H]^−^, and 359 [2 M–C_18_H_16_O_8_–H]^−^. Moreover, compound 18 ([M–H]^−^, *m*/*z* 137) was tentatively determined as 4‐hydroxybenzoic acid because of its fragment ions *m*/*z* 93 [M–COO–H]^−^. The compound 15 ([M–H]^−^, *m*/*z* 169) showed a single fragment peak at *m*/*z* 125 indicated the presence of trihydroxy phenol moiety and the loss of ‐COO. Consequently, it was tentatively detected as gallic acid. Additionally, the 4‐hydroxybenzoic acid 1‐O‐β‐D‐glucopyranosyl ester and 4‐hydroxybenzoic acid were identified in free phenolics extracted from okra fruit (D'Urso et al. 2020), while rosmarinic and gallic acid were identified in okra leave (Mohammadi et al. 2021). Compound 16 ([M–H]^−^, *m*/*z* 153) was tentatively characterized as 3,4‐dihydroxybenzoic acid, supported by its fragment ions at *m*/*z* 108 [M–CO_2_H–H]^−^, 109 [M–CO_2_–H]^−^, and 125 [M–CO–H]^−^. Compound 17 ([M–H]^−^, *m*/*z* 177) showed two fragment peaks at *m*/*z* 133 and 105 because of the COO losing and subsequent loss of CO. Based on its distinctive features, compound 17 was tentatively identified as esculetin. In conclusion, the constituents of OPBP and OSBP differed, and only quercetin and luteolin 4′‐O‐glucoside were identified in both OPBP and OSBP.

### 3.3 In Vitro Antioxidant Activities of OPBP and OSBP

**FIGURE 4:**
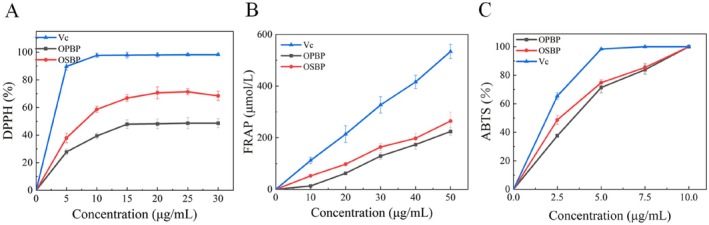
The antioxidant activity of OPBP and OSBP in vitro: DPPH (A), FRAP (B), and ABTS (C). Data points represent means ± standard deviations (*n* = 3).

The antioxidant activity of OPBP and OSBP was comprehensively evaluated based on FRAP, DPPH, and ABTS assays for the first time. Usually, the hydrogen‐donating capacity of phenolic compounds is evaluated by assay of DPPH radical scavenging. The highest DPPH radical scavenging rate of OSBP was 78% higher than that of OPBP (47%), while they were lower than that of Vc (Figure 4A). The DPPH radical scavenging ability of OPBP was found to be lower than that of free polyphenols extracted from okra pulp (Wang et al. 2023). The FRAP value of OPBP and OSBP enhanced in dose‐dependent (Figure 4B). The FRAP values of OPBP (4.30 mmol Fe^2+^/g) and OSBP (5.18 mmol Fe^2+^/g) were significantly higher than that of free phenolics extracted from okra pulp (1.43 mmol Fe^2+^/g) and okra seed (0.76 mmol Fe2^+^/g) (Xia et al. 2015), while lower than that of free phenolics extracted from the okra pulp (9.77 mmol Fe^2+^/g) (Wang et al. 2023). These discrepancies may be attributed to differences in okra cultivars, growing conditions, and extraction methods. When their concentration reached 10 μg/mL, both OPBP and OSBP exhibited a clearance rate of 100% for ABTS (Figure 4C). The IC_50_ values of OPBP and OSBP were 3.34 and 3.69 μg/mL, respectively. The ABTS IC_50_ value of methanolic extract from *Empetrum rubrum* Vahl ex Willd was 0.1088 mg/mL (Schneider et al. 2024). The ABTS IC_50_ values of the maceration, ultrasound, and reflux heating extracts from *Anvillea garcinii subsp. radiata* were 21.50, 23.85, and 29.24 μg/mL, respectively (Oucheikh et al. 2026). These results also indicated that both OPBP and OSBP demonstrated excellent ability to clear ABTS. The OSBP shows higher FRAP and DPPH compared with OPBP, while it shows lower ABTS. It can be attributed to the fundamental differences of their reaction mechanisms and the specific chemical structures of the phenolic profile. The high FRAP value of OSBP suggests a high concentration of electron‐donating phenolics, likely facilitated by its presence of methoxylated flavonoids and aglycones. These compounds often have lower redox potentials, making them potent reducers of ferric ions. The DPPH assay is conducted in ethanol environment, whereas ABTS can be performed in aqueous buffer. The active phenolics in OSBP may have lower solubility or slower diffusion rate in the standard ABTS solution, leading to its lower ability to clear ABTS. This highlights that the antioxidant capacity is not absolute but depends on the chemical context of the assay.

### 3.4 The Antibacterial Activity of OPBP and OSBP

#### 3.4.1 The Effect of OPBP and OSBP on Bacterial Vitality

**FIGURE 5:**
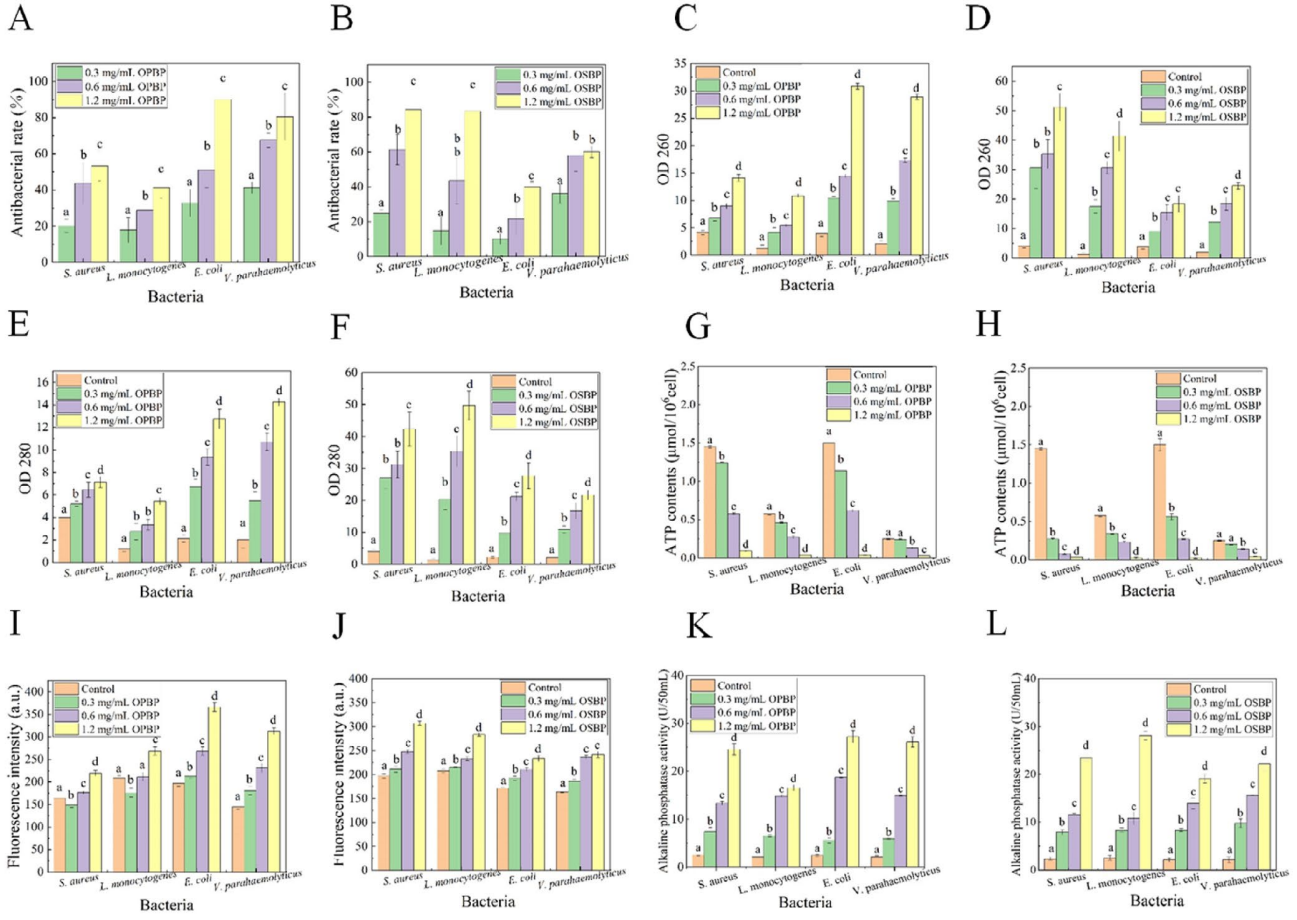
Antibacterial rate (A), OD 260 (C), OD 280 (E), ATP content (G), ROS formation (I), and alkaline phosphatase activity (K) of OPBP; antibacterial rate (B), OD 260 (D), OD 280 (F), ATP content (H), ROS formation (J), and alkaline phosphatase activity (L) of OSBP. Data points represent means ± standard deviations (*n* = 3). Different lowercase letters indicate statistically significant differences (*p* < 0.05).

We originally planned to freeze‐dry OPBP and OSBP to prepare higher concentration solutions. However, due to their extremely strong hygroscopicity, both substances rapidly absorbed moisture upon exposure to air and turned into viscous liquids, making accurate weighing unfeasible. Therefore, a reduced‐pressure concentration method was used to obtain different concentrations of OPBP and OSBP. Nevertheless, the maximum concentration achievable through this method was limited to 1.2 mg/mL. As shown in Figure 5A,B, OPBP (1.2 mg/mL) exhibited stronger antibacterial activity against Gram‐negative bacteria, showing the highest inhibition rate of 87.74% ± 7.73% against *E. coli* and 78.19% ± 8.64% against *V. parahaemolyticus* . However, its highest inhibition rate against *L. monocytogenes* and *S. aureus* was only 40.31% ± 8.65% and 53.31% ± 5.78%, respectively. As for OSBP, it showed more than 80% inhibition against *S. aureus* and *L. monocytogenes* , which was obviously higher than that against *E. coli* and *V. parahaemolyticus* . The different inhibition activities of OPBP and OSBP against bacteria may be attributed to their distinct phenolic compositions. It is well known that the cell wall structure of Gram‐negative and Gram‐positive bacteria is significantly different. The cell walls of Gram‐positive bacteria are thick and compose of peptidoglycan and acidic polysaccharides. Phenolic compounds can destroy their cell wall by linking to peptidoglycan, resulting in bacterial death. However, the peptidoglycan layer of Gram‐negative bacteria cytoderm is thin, and differently, it contains an additional outer membrane preventing polyphenols from linking to peptidoglycan. The outer membrane is mainly composed of lipopolysaccharides, lipids, and lipoproteins. The higher inhibition rate of OPBP against *E. coli* and *V. parahaemolyticus* may be explained by its abundance of phenolic compounds such as eupatilin, oroxylin A, hesperetin, and formononetin. These compounds contain hydrophobic groups (OCH_3_ or C_5_H_8_) which likely facilitate penetration through the outer membrane. Bai et al. (2023) reported that the minimum inhibitory concentration (MIC) of biocatechin A was 128 μg/mL against *S. aureus* ATCC29213. Additionally, the stem bark methanol extract (1.0 mg/mL) possessed the rate of 60% for preventing the growth of *E. coli* and *Bacillus cereus* (Son et al. 2018). Besides, the concentrations tested of OPBP and OSBP for antibacterial activity are relatively high compared to antibiotic MICs (Hanci and Igan 2023), limiting their direct application as a primary antimicrobial. Consequently, the realistic applications of OPBP and OSBP could rather be as complementary preservatives or components of active packaging/combined systems. It should be noted that the current study evaluated relative growth inhibition rates. Future work will determine precise MIC values and include antibiotic controls to strictly benchmark efficacy according to Clinical and Laboratory Standards Institute (CLSI) standards.

#### 3.4.2 Effects of OPBP and OSBP on Release of Nucleic Acid and Protein

The cell membrane is not only a structural component, but also an important protective barrier of the cell. Phenolic compounds, owing to their benzene ring and the hydroxyl functional groups, could disrupt the cell membrane of bacteria. Such damage could result in the leakage of cellular components such as proteins and nucleic acid, which can be used as an indicator to evaluate the structural integrity of the cell membrane. Comparing with the control, the OPBP and OSBP significantly increased the membrane permeability of four kinds of bacteria (Figure 5C–F). The higher the concentration of OPBP and OSBP, the greater the permeability of the cell membrane, aligning with their antibacterial rate.

#### 3.4.3 Effect of OPBP and OSBP on Intracellular ATP Content

Under normal condition, the intracellular ATP content is stable, while the inhibitor can disrupt the cell membrane, resulting in the decreasing of ATP synthesis and a consequent reduction in intracellular ATP. All tested concentrations (0.3, 0.6, and 1.2 mg/mL) of OPBP and OSBP resulted in lower ATP levels in bacterial cells compared to the control group (Figure 5G,H). Furthermore, the ATP content decreased progressively with increasing concentrations of OPBP (or OSBP). This finding provides further evidence that OPBP and OSBP compromise cell membrane integrity, thereby affecting normal cellular activities. Similar decreasing of intracellular ATP level was also reported in polyphenols extracted from other plants (Wang et al. 2024; Rui et al. 2021).

#### 3.4.4 Effects of OPBP and OSBP on ROS Generation

Although the antioxidant or pro‐oxidant properties of polyphenols are based on their chemical structure, the environmental factors (high pH, high concentration, and transition metal ion) could also induce pro‐oxidant activity in otherwise antioxidant compounds (Dong et al. 2021). The fluorescence intensity of 2′, 7′‐dichlorofluorescein is directly proportional to the amount of ROS because H_2_DCF (production of H_2_DCFDA) is able to generate 2′, 7′‐dichlorofluorescein when ROS exists. In this study, the fluorescence intensity in bacteria was generally higher than that of the control and increased in a dose‐dependent manner with increasing concentrations of OPBP and OSBP. The only exception was observed in *S. aureus* and *L. monocytogenes* treated with 0.3 mg/mL OPBP, which showed lower fluorescence intensity than the control (Figure 5I,J). This concentration‐dependent shift from antioxidant to pro‐oxidant activity aligns with previous findings reported by Banerjee et al. (2008) for curcumin. Generally, the ROS generated by the stimulation of phenolic compounds is beneficial to their antibacterial activity. Inhibiting growth and reproduction of bacteria through inducing ROS generation by phenolic compounds was also reported in other research (Liu et al. 2023). The ROS could trigger oxidative stress, leading to DNA degradation, protein denaturation, and membrane disruption, ultimately resulting in cell death. However, in this study, we present this cautiously that the induction of oxidative stress might contribute to the antibacterial effect. As the H_2_DCFDA assay provides a general marker of oxidative stress but does not prove that ROS generation is the primary or sole mechanism of lethality.

#### 3.4.5 Effect of OPBP and OSBP on Alkaline Phosphatase Activity in Bacteria

The leakage of alkaline phosphatase in a bacterial suspension can reflect the integrity of the cell wall. This is because alkaline phosphatase only exists between cell wall and cell membrane. Comparing to control group, treatment with OPBP (or OSBP) significantly increased the alkaline phosphatase activity in the suspensions of bacteria (*p* < 0.05) (Figure 5K,L). Furthermore, the leakage of alkaline phosphatase activity exhibited a clear dose‐dependent relationship with increasing concentrations of OPBP (or OSBP). This indicates that OPBP and OSBP increase the permeability of the cell wall. This phenomenon was also reported by Wang et al. (2024), who studied the bacteriostatic activity of polyphenol extracts from flaxseed against *Pseudomonas fluorescens* . Further, other mechanisms (e.g., metal chelation, interference with key metabolic enzymes) were not examined in this study and may contribute to the antibacterial ability of OPBP and OSBP.

#### 3.4.6 The Morphology Changes of Bacteria

**FIGURE 6:**
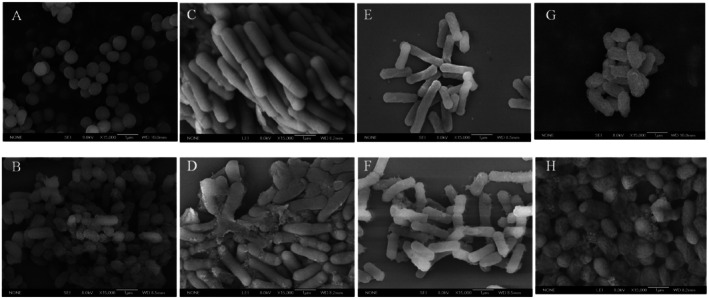
The morphology changes of bacteria: *S. aureus* and *V. parahaemolyticus* before (A, C) and after OPBP treatment (B, D); *E. coli* and *L. monocytogenes* before (E, G) and after OSBP treatment (F, H).

The untreated *S. aureus* cells showed regular spherical morphology with a smooth surface, uniform size, and homogeneous distribution (Figure 6A). After treated with OPBP, the cellular morphology of *S. aureus* changed significantly, and some cell lysis was observed (Figure 6B). Besides, the cell edges of *S. aureus* appeared stinging protuberance. No obvious cell lysis was observed in *E. coli* treated with OSBP, while a layer of polymer similar to tiny particles was attached to its surface (Figure 6F). Expectedly, as for *V. parahaemolyticus* treated with OPBP and *L. monocytogenes* treated with OSBP, their cells were damaged obviously (Figure 6D,H). Furthermore, while the data focuses on membrane integrity and oxidative stress, we acknowledge that other mechanisms may also contribute to the overall antibacterial activity. Potential interactions, such as metal chelation by the test compound or specific inhibition of vital enzymatic targets, were not investigated in this study. These alternative or synergistic pathways warrant further investigation in future work.

Although the in vitro results are promising, several limitations must be acknowledged. The bioactivity demonstrated in this study is based solely on in vitro models. The actual bioavailability, absorption, and metabolic fate of these bound phenolics in a living organism remain unknown. In vivo studies are required to confirm whether the observed antioxidant and antimicrobial effects translate to physiological conditions. Furthermore, the impact of gastrointestinal digestion on the structure and activity of these phenolics was not assessed. Future studies should verify physiological efficacy through in vivo experiments and evaluate structural stability and bioaccessibility using simulated digestion models.

## 4 Conclusion

The maximum TPC of OPBP and OSBP were 7.77 and 83.03 mg GAE/g DW, respectively. Then 14 phenolic compounds in OPBP and 12 phenolic compounds in OSBP were identified by HPLC‐ESI‐QTOF‐MS/MS. Unlike free phenolics extracted from okra, there are few dimer and flavonoid glycosides in bound phenolic fractions. Besides, only quercetin and luteolin 4′‐O‐glucoside were identified in both OPBP and OSBP indicating distinct constituents of bound phenolics in pulp and seed. Compared with OPBP, the OSBP showed higher antioxidant activity. Furthermore, OPBP exhibited stronger antibacterial activity against *E. coli* and *V. parahaemolyticus* , while OSBP was more effective at inhibiting the growth of *S. aureus* and *L. monocytogenes* . To our knowledge, this study provides the first exhaustive profile of bound phenolic compounds in okra. The OPBP and OSBP exerted antibacterial activity and antioxidant capacity, suggesting that they may be further explored as complementary natural preservative components for specific products, or as functional ingredients in plant‐based foods. Additional, future work should include simulated digestion to assess bioaccessibility and in vivo models to verify antioxidant and antibacterial efficacy in food or biological systems.

## Author Contributions

**Rongjuan Liu:** data curation, formal analysis, investigation, visualization, writing – review and editing. **Haiqiang Chen:** formal analysis, visualization. **Xiaolin Shi:** resources, project administration. **Yinping Li:** conceptualization, funding acquisition, supervision, writing – original draft, writing – review and editing, validation. **Ting Xv:** data curation, formal analysis, visualization, writing – original draft. **Hong Zhang:** data curation, investigation, visualization, writing – review and editing. **Mingyan Yan:** project administration, supervision. **Yuan Xv:** formal analysis, visualization. **Hu Zhu:** software, supervision. **Bingjie Zhang:** conceptualization, methodology, formal analysis, writing – original draft, writing – review and editing, data curation, investigation, validation. **Yue Lv:** data curation, formal analysis, writing – original draft.

## Funding

This work was supported by Natural Science Foundation of Shandong Province, ZR2022MC101.

## Ethics Statement

The authors have nothing to report.

## Conflicts of Interest

The authors declare no conflicts of interest. Yuan Xv is an employee of Shandong Nice Health Technology Co. LTD. Haiqiang Chen and Hu Zhu are employees of Shandong Guangpu Biotechnology Co. Ltd. The two companies did not participate in experiment design, data curation, analysis, decision to publish of the manuscript. The remaining authors declare that the research was conducted in the absence of any commercial or financial relationships that could be construed as a potential conflict of interest.

## Data Availability

The data supporting the findings of this study are available from the corresponding author upon reasonable request.
